# Occult HBV Infection: A Faceless Enemy in Liver Cancer Development

**DOI:** 10.3390/v6041590

**Published:** 2014-04-08

**Authors:** Jaime Morales-Romero, Gustavo Vargas, Rebeca García-Román

**Affiliations:** Instituto de Salud Pública, Universidad Veracruzana, Av. Luis Castelazo Ayala s/n Col. Industrial Ánimas, 91190, Xalapa, Veracruz, Mexico; E-Mails: jaimemrom@gmail.com (J.M.-R.); gvargas@uv.mx (G.V.)

**Keywords:** occult hepatitis B infection, hepatocellular carcinoma, hepatitis B virus

## Abstract

The hepatitis B virus (HBV) represents a worldwide public health problem; the virus is present in one third of the global population. However, this rate may in fact be higher due to occult hepatitis B virus infection (OBI). This condition is characterized by the presence of the viral genome in the liver of individuals sero-negative for the virus surface antigen (HBsAg). The causes of the absence of HBsAg in serum are unknown, however, mutations have been identified that produce variants not recognized by current immunoassays. Epigenetic and immunological host mechanisms also appear to be involved in HBsAg suppression. Current evidence suggests that OBI maintains its carcinogenic potential, favoring the progression of fibrosis and cirrhosis of the liver. In common with open HBV infection, OBI can contribute to the establishment of hepatocellular carcinoma. Epidemiological data regarding the global prevalence of OBI vary due to the use of detection methods of different sensitivity and specificity. In Latin America, which is considered an area of low prevalence for HBV, diagnostic screening methods using gene amplification tests for confirmation of OBI are not conducted. This prevents determination of the actual prevalence of OBI, highlighting the need for the implementation of cutting edge technology in epidemiological surveillance systems.

## 1. Introduction

Liver cancer is considered to be a global health problem and is the second most frequent cause of cancer mortality worldwide. The main risk factor for liver cancer development is the hepatitis B virus (HBV) [1]. Hepatocellular carcinoma (HCC) is the main tumor produced in the liver, and is directly associated with the prevalence of HBV infection [2]. According to World Health Organization (WHO), worldwide there are two billion people with positive serological markers for HBV [3] of which more than 240 million are chronically infected [4]. A third of the global population has been exposed to HBV [5]. In 2012, it ranked as the second leading cause of cancer death worldwide after lung cancer [6] causing about 600,000 deaths yearly [4].

Chronic infection can be acquired just after birth, and is implicated in the development of the majority of HCC [7]. The incidence of HCC in HBV endemic areas, such as East Asia and sub-Saharan Africa, tends to be high as a result of this chronic neonatal infection [8]. Across the world, the incidence is considered from moderate to high risk area for contracting HBV infection; for example, Latin America has a prevalence of infection lower than 3% [9]. Nevertheless, the true prevalence of HBV could be underestimated due to the presence of a poorly characterized condition known as occult HBV infection (OBI). This infection is described as the presence of HBV DNA in the serum or liver, in the absence of detectable levels of hepatitis B surface antigen (HBsAg) in the serum. Moreover, the non-systematic epidemiological surveillance mechanisms that are established in Latin American countries such as Mexico employ outdated diagnostic methodologies. In this review, we explore some of the currently described mechanisms involved in the development of OBI, and its involvement in liver carcinogenesis. The review also includes a brief description of the epidemiological implications of underreporting and inappropriate treatment of OBI in Latin American countries such as Mexico, where the low reported values of incidence do not reflect the true prevalence of infection.

## 2. The Hepatitis B Virus

The hepatitis B virus is an enveloped DNA virus that belongs to the family Hepadnaviridae and features partially double-stranded relaxed-circular DNA. There are eight genotypes of HBV, classified from A to H, and these are distributed across different geographic zones. The entire genome is 3.2 kb in length and replicates by reverse transcription of an intermediate known as pregenomic RNA (pgRNA). The infectious viral particle is spherical and of 40–45 nm in diameter. The virus consists of an inner nucleocapsid or core, surrounded by a lipid envelope containing virally encoded surface proteins [10].

The genome of HBV is divided into four main regions that are fragments of open reading frames (ORFs) containing four overlapping genes: C/PreC, S/PreS, X and Pol, controlled by four promoters. The ORF C/PreC partially overlaps with ORF P and encodes the precore-core protein (HBeAg) and core protein (HBc) [11]. HBeAg seropositivity is a marker for active viral replication. In the natural history of chronic hepatitis B infection, HBeAg marks the first two of the four phases, namely the immune tolerant phase and the immune clearance phase, and is associated with highly replicative activity of the HBV [12].

The S/preS gene codes for three envelope proteins that are generated from three different start codons. The large surface protein (L) is the translation product of the whole open reading frame (400 amino acid residues for HBV genotype A). The middle (M) lacks the N-terminal 119 aa of L (the pre-S1 sequence), and the small (S) lacks the N-terminal 55 aa of M (the pre-S2 sequence) [13]. The HBsAg consists of S (S, small surface), MS (medium surface, S+preS2), and LS (large surface, S+preS1+preS2) HBsAg molecules [14]. The three proteins are post-translationally modified: each of them exhibits a partially glycosylated site in the S domain, an additional glycosylation in the pre-S2 region of the M protein, and the L protein differs from the other envelope proteins by a N-terminal myristylation [15]. HBsAg particles derived from human plasma or HBsAg particles produced by recombinant DNA methods (some of which lack pre-S epitopes) have been shown to elicit a protective immune response, and the purified particles represent current vaccines for HBV [16,17]. For the development of these vaccines, the preS1 sequence of HBsAg has been shown to be a particularly efficient immunogen at T-and B-cell levels. Five distinct antibody-binding sites within the preS1 region of HBsAg/P43 of the *adw* subtype: preS1 (16–27), preS1 (32–53), preS1 (41–53), preS1 (94–105) and preS1 (106–117) have been determined in mice [18,19,20]. Third generation HBV vaccines containing Pre-S1 and Pre-S2 antigens have been developed with reported excellent immunogenicity in humans, including rapid onset of antibody responses towards the S-protein of the vaccine [21,22].

## 3. Occult HBV Infection

Infection by HBV is divided into five clinical categories: asymptomatic, acute, chronic, fulminant and OBI [23]. The latter was defined at an international workshop in Italy in 2008 [24] as the “presence of HBV viral DNA in the liver (with or without detectable HBV DNA in serum) of HBsAg-negative individuals tested with the currently available serum assays”. A cutoff value of <200 IU/mL was also introduced for HBV DNA in serum. From the early 1980s, it was suspected that HBV could persist undetected in the host [25], but this became more evident when patients who received blood transfusions from HBsAg-negative donors went on to develop the open infection. 

Epidemiologically, people at high risk of becoming infected with hepatitis B virus could be more likely to develop OBI; however, since testing for the virus is not always conducted systematically in these groups, there is a lack of epidemiological behavior in health institutions. Previous studies have focused on certain groups of interest, including hemodialysis patients infected with human immunodeficiency virus, hemophiliacs, hepatitis C infections, certain ethnic groups and apparently healthy blood donors.

For example, Table 1 presents the reported global prevalence of OBI in hemodialysis patients and in those infected with human immunodeficiency virus (HIV). There is a notable variation in the prevalence of OBI in patients on hemodialysis, ranging from 0–58% [26,27,28,29,30,31,32]. Similarly, OBI frequencies in HIV-infected subjects ranged from 0–89.5% [33,34,35,36,37].

In eastern countries, OBI has been reported in 0.1%–2.4% of blood donors. This pattern has also been shown in the United States, where only 5% of the population has been exposed to HBV [38]. In the Asian population, however, OBI prevalence is much higher and ranges from 7.5%–16% [39]. In several groups, the prevalence of OBI would be even greater were the tests to be performed in liver tissue. Another report has shown the presence of OBI in 45%–50% of intravenous drug users or patients with hemophilia, in up to 36% of patients undergoing hemodialysis [29], 8%–51% of patients with HIV [40] and in 30%–95% of HBsAg-negative patients with chronic hepatitis C [41].

Prevalence of OBI varies according to geographical region, but also depends greatly on the specificity and sensitivity of the routine serological assays or nucleic acid testing (NAT). Moreover, patients included in testing for OBI often-present different inclusion criteria, and therefore it is not always possible to compare them.

**Table 1:** Prevalence of occult hepatitis B virus infection (OBI) in hemodialysis and human immunodeficiency virus (HIV) patients.

Author (Publication year) (Reference)	Population	*n*	Prevalence of anti–HBsAg	Prevalence of anti–HBc	Prevalence of occult HBV infection	Comments
Hemodialysis patients						
Cabrerizo M, *et al.* (1997) [26]	Spain	33	14 (42%)	14 (42%)	19 (58%)	
Besisik F, *et al.* (2003) [27]	Turkey	33			12 (36.4%)	All patients had HCV infection.
Abu El Makarem MA, *et al.* (2012) [28]	Egypt	145	15 (10.3%)	29 (20%)	6 (4.1%)	Patients with or without HCV infection were included.
Minuk GY, *et al.* (2004) [29]	Canada	239	152 (63%)	21 (8.7%)	9 (3.8%)	
Albuquerque AC, *et al.* (2012) [30]	Brazil	752	201 of 752 (26.7%)	135 of 201 (67.2%)	3 of 201 (1.5%)*	
Fabrizi F, *et al.* (2005) [31]	Italy	213	120 of 316 (37.9%) ^†^	123 (57.7%)	0 (0%)	Patients undergoing hemodialysis or peritoneal dialysis were included.
Goral V, *et al.* (2006) [32]	Turkey	50	21 (42%)	4 (8%)	0 (0%)	
HIV+ patients						
Hofer M, *et al.* (1998) [33]	Switzerland	57	0 (0%)	56 (98.2%)	51 (89.5%)	Longitudinal observation
Marite B, *et al.* (2011) [34]	Cube	325	45 of 99 (45.5) ^‡^	99 (30.5%)	13 of 54 (24.1%) ^§^	
Filippini, *et al.* (2006) [35]	Italy	115	16 of 115 (13.9%) (baseline)	58 of 115 (50.4%) (baseline)	17 of 86 (19.8%) ^||^	Longitudinal design
Panigrahi R, *et al.* (2012) [36]	India	112			12 of 112 (10.7%)	
Nunez M, *et al.* (2002) [37]	Spain	85	Not reported	Not reported	0 (0%)	

HCV: Hepatitis viral C; Inclusion criteria: * HBV DNA was searched only in 201 anti–HBc positive patients; ^†^ Anti HBsAg was detected in a subset of patients available; ^‡^ Only anti–HBc positive patients were tested; ^§^ Only anti–HBc positive patients with anti–HBs levels of <50 IU/L were tested. ^||^ Only 86 patients were followed up.

## 4. Principal Molecular Mechanisms Associated with OBI

There have been numerous studies that attempt to elucidate the mechanisms involved in the development of OBI. Some proposals from these studies attempt to explain the persistence of HBV DNA in HBsAg-seronegative immunocompetent individuals. These proposals range from viral genome integration in the host chromosomes, mutations in the region of the S gene helix (undetected in diagnostic tests), the window period after acute infection, immunosuppression of the host, HCV co-infection competing with HBV, low capability for detection of HBsAg in laboratories and serology kits with low specificity and sensitivity to HBsAg, among others [42]. One of these studies showed a prevalence of 17% in OBI liver samples from people with no apparent liver disease [43]. This indicates that the host immune response remains the viral infection under control with no apparent clinical manifestations. Another study characterized the response of T lymphocytes specific to HBV in OBI patients, and found that HBsAg-seronegative, but HBcAg-positive patients showed a typical memory response in the T lymphocytes [44]. However, other explanations are proposed for the origin of OBI, such as mutations in transcription-controlling regions of the polymerase domain that lead to a decrease in viral replication and low or zero expression of HBsAg [45]. Co-infection with HCV or hepatitis delta virus (HDV) has also been reported in patients with OBI, promoting low HBV replication and reducing the synthesis of HBsAg to undetectable levels in the serum [46].

### 4.1. Genome Integration of HBV

Although the specific mechanisms that cause OBI are still not fully understood, several studies have suggested the possible origins of this condition. Prolonged persistence of covalently closed circular DNA (cccDNA) in the hepatocyte nucleus has been reported in carriers of occult HBV [47]. The average number of copies of genetic material per hepatocyte has been estimated at approximately 1.5, but ranges from <0.01 to >50 copies/cell [48]. The virus life cycle is a fundamental step for the establishment of OBI. The cccDNA is an intermediate replicative form that persists in the cell nucleus as chromatinized episomal DNA, which is very stable and serves as template for gene transcription [49]. Both the stability and persistence of cccDNA, as well as the long half-life of the hepatocyte, mean that HBV infection could persist for the entire life of the host [50]. In addition, episomal HBV DNA has been found integrated within the host chromosomes of individuals with HCC [51]. The integration of HBV is partial, and involves only certain gene sequences rather than the entire genome [52]. Indeed, the HBV core gene is lost during integration of the genome, which prevents expression of the protein core and thus serves as a sentinel for the diagnosis of infection [53].

### 4.2. Genetic Mutations in HBV

**Figure 1:**
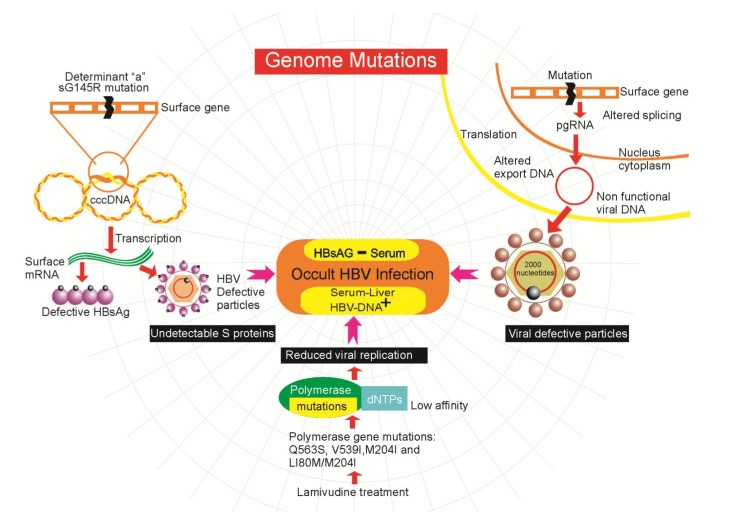
Genome mutations associated with the development of occult hepatitis B virus infection (OBI).

The OBI seems to be mainly linked to a strong suppression of viral replication, genome mutations [54], genetic expression and secretion of the virus, rather than a high capacity for integration of its genome. The low levels of replicative activity may be the result of the presence of defective particles as a result of mutations in the transcription control regions or polymerase domains leading to inefficient replication in conjunction with a dissonant release of HBsAg by the hepatocytes [46]. Many reports have found mutations and rearrangements in the donor splice sites of the pre-S1, Pres-S2/ and S genes in patients with OBI [55]. These mutations may be associated with reduced expression and secretion of HBsAg and could affect pre-S2/S mRNA splicing [56]. Other mutations found in the overlap region of the core gene promoter, influence the low replicative potential while variants in the Pre-Core/Core sequences reduce the efficiency of replication through the functional structure of the epsilon signal, which is essential for pregenomic encapsidation and initiation of HBV DNA synthesis [57]. Mutations in the “a” determinant region of the surface gene are frequently found in samples taken from patients with OBI and different liver diseases. The sG145R mutation was one of the first to be described and explains the molecular mechanisms that lead to OBI (Figure 1). Modification in the protein as a result of this mutation prevents its detection by immunoenzymatic assay [24]. The mutant sG145R shows a low affinity for the monoclonal antibody that recognizes HBsAg in diagnostic tests. Furthermore, substitutions have been found in the most hydrophilic region (MHR) of the S gene that alter the antigenicity of HBsAg and/or the infectivity of the virus [58]. 

Also, a series of mutations caused by treatment with lamivudine have been described, which are used to combat viral infections. This drug causes changes in the polymerase and surface genes. The mutations Q563S, V539I, M204I, and L180M/M204I confer resistance to lamivudine therapy and reduce the affinity of the polymerase to its natural substrates, the dNTPs (Figure 1), leading to lower viral replication [59]. Even a reduction in the binding antibody to a range of S mutants derived from commonly selected lamivudine-resistant HBV mutants has been shown. The expressed proteins containing these mutations had an altered antigenicity and may have the potential to escape neutralization by anti-HBs antibody [60,61]. This is relevant because in another study six of 12 patients with OBI showed HBV DNA mutations associated with lamivudine resistance, even though none of the patients had undergone lamivudine therapy [60,61]. This is more than relevant due to the potential transmission of lamivudine resistant HBV in blood transfusion. It is important to note that people with open HBV infections that receive treatment with lamivudine may be selecting mutants that escape detection in commercial assays, which may eventually lead to a chronic occult infection. However, the real effect of lamivudine resistance mutations on HBV replication during OBI remains unknown.

Another mutation in the surface gene (G458A) can alter mRNA splicing of the gene, affecting exportation of mRNA and DNA folding, leading to zero expression of HBsAg [62]. The participation of the S gene is important to the viral infectivity of HBV, since it is known that high levels of the S gene promote the assembly and secretion of excessive amounts of noninfectious HBsAg particles from the cell, independently of the release of the virion. Alteration of splicing by mutations in the genome may also interfere with viral replication via the pgRNA. Splicing of the pgRNA produces a non-functional viral DNA of reduced size (from 3100–2000 nucleotides) that can, however, encapsidate and export to the medium [63].

In addition, an alternative RNA splicing event has been reported (reduction of 2986 nucleotides to 202) that suppresses expression of the surface protein gene without affecting the polymerase and functions related to the core or X proteins. This splicing generates intracellular virus particles, without the surface protein, that subsequently accumulate mutations due to the relaxation of restrictions on encryption. Such viruses are deficient in terms of autonomous propagation and cannot leave the host cell until it is lysed [64].

### 4.3. Epigenetics Mechanisms

Methylation of HBV DNA is another mechanism that has been explored as a cellular defense mechanism for silencing viral genomes. It has been found that methylation of cytosines in CpG dinucleotides in the promoter regions of genes leads to gene silencing [65]. The HBV genome of 3.2 kb double stranded circular DNA contains three CpG-rich regions spanning the ATG site for the surface antigen gene, the promoter of the gene for protein X and the ATG site of the polymerase. It has also been observed that when DNA constructs of the methylated HBV are transfected *in vitro* in hepatocyte cell lines, expression of HBsAg is reduced by more than 90% [66]. Methylated HBV sequences have even been found integrated into the host genome in samples of patients with HCC associated with OBI [66,67]. However, it remains unclear whether the cellular machinery utilizes DNA methylation to silence the HBV genome as a form of self-defense or whether the HBV genome takes advantage of cellular methylation to escape detection by the immune system of the host [68].

The acetylation of histones bound to the DNA is another mechanism that regulates the transcriptional activity of HBV. Hyperacetylation of histones bound to cccDNA has been associated with increased viral replication in cell culture [53]. A mutant HBx of only one nucleotide has been associated with a rapid hypoacetylation of histones bound to cccDNA, thereby weakening the recruitment of the p300 transcriptional activator [69].

### 4.4. Host Immune Response in OBI

While the molecular basis of the origin of OBI, involving alterations at the DNA and RNA level, offers an insight into the etiology of this condition, the role of the host immune response in containing the infection cannot be disregarded. It is presumed that humoral and cellular immunological pressure on the HBV coat proteins are principal mechanisms in the generation of OBI [70]. This specific immunological pressure against HBV may contribute to the development of OBI and explain why reactivation of infection is observed under immunosuppression [25]. It has been reported that acute hepatitis can occur following restoration of the immune system after immunosuppressive treatment for hematopoietic cancers, and following organ and stem cell transplants [71].

**Figure 2:**
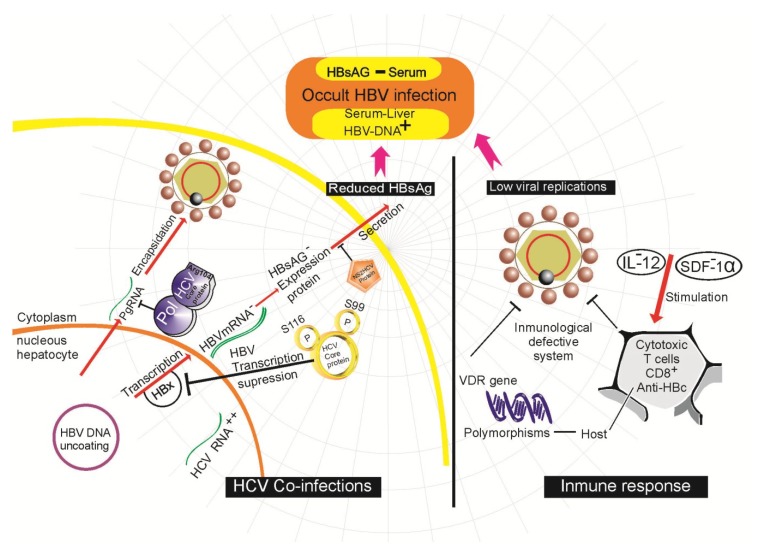
Molecular mechanisms described in occult hepatitis B virus infection (OBI) development.

Previous studies show that anti-HB-c positive patients show a response typical of protective memory T lymphocytes, suggesting that this condition represents a resolved infection with immune-mediated virus control. In contrast, no HBV-specific T lymphocyte response has been observed in HB-c negative patients, raising the possibility that a low level viral infection could be insufficient to permit maturation of the protective immunological memory [46]. Suppression of viral transcription has also been observed during HBV clearance that suppresses replication, resulting in HBsAg negativity and low or undetectable serum levels of HBV DNA in the presence of intrahepatic HBV DNA [46]. In a study in Mexico, the expression profiles of different pro- and anti-inflammatory cytokines were analyzed for the first time in indigenous OBI patients infected with genotype H. This study reported an elevated increase in serum levels of transforming growth factor-beta (TGF-beta) and overexpression of interleukin-2 (IL-2) found exclusively in patients with OBI [72]. Moreover, another study showed a similar response in terms of overexpression of interleukins IL-10, IL-17 and IFN-gamma, while IL-12 and stromal cell-derived factor (SDF)-1alpha presented no difference in OBI patients when compared to healthy subjects who were clear of HBV infection [73,74,75]. Patients with OBI seem to be unable to express high levels of IL-12 and SDF-1alpha, a fact that could facilitate clearing of HBV through activation and migration of T lymphocytes and NK cells [76] (Figure 2). A related study found a decrease in CD8+ T cells in individuals with OBI, compared to healthy individuals [76]. This is very important because both T lymphocytes and NK cells are the most important cells of the immune system against viral infections, and their numbers increase during the acute phase of infection.

### 4.5. Viral Co-Infection

Co-infection with HCV and HBV is another factor involved in the genesis of OBI. It has been shown that the rate of clearance of HBsAg is 2.5 times higher in HCV/HBsAg positive cases than in HCV infection only, suggesting that HCV is the most important hepatotropic virus in terms of enhancing the clearance of HBsAg in chronic hepatitis B [77]. This has been clearly observed in recipients of blood transfusions infected with HCV and HBV, where the initial appearance of HBsAg is often delayed and subsequently followed by an interval of detection of HBsAg and a reduction in the peak levels of HBV DNA [78]. Indeed, an inverse correlation exists between HCV RNA and HBV DNA, even within the same hepatocyte. Related studies show the presence of 1.6 times more DNA in hepatocytes infected with HBV alone, compared to those infected with both HCV and HBV [79]. One of the mechanisms that attempt to explain how HCV can inhibit the replication of its competitor virus (HBV) has been the role of the HCV core protein. This protein is required for the suppressor activity of HCV on the replication of HBV as well as the expression of HBsAg. Phosphorylations at serine 116 and serine 99 are required for the suppressive activity of the core protein. The protein binds to the HBV polymerase and can thus prevent binding of the polymerase to its packaging sequence (Figure 2), preventing transcription of the HBV mRNA and encapsidation of the pgRNA [80]. In fact, arginine 104 of the HCV core protein inhibits HBV encapsidation by binding to the polymerase without affecting HBV gene expression [80]. The NS2 protein of the HCV (Figure 2) is another involved in the inactivation of HBV. This protein exhibits proteolytic activity; when co-transfected in plasmids with HBV dimers, inhibition of secretion of HBsAg and HBeAg toward the supernatant is observed, along with inhibition of HBV replication [81].

### 4.6. Other Mechanisms

Recent research has indicated that the Vitamin D receptor (VDR) plays a key role in host immune response. Both Vitamin D3 and its receptor regulate the expression of various cytokines and are important determinants in the response against HBV [82]. It has even been shown that functional polymorphisms in the region of the intron 8 and the 5’ of exon 9 can influence expression of the VDR gene in patients with OBI (Figure 2) [83]. However, there are no additional data that can support and confirm these studies, so they should be taken with caution.

As stated previously, several mechanisms have been proposed that, either alone or in unison, participate in the development of OBI. This can be through alterations to the infectious agent itself (genomic mutations), through competition with other hepatotropic agents or through the host response to infection. This ultimately leads to zero detection of HBsAg in the serum of patients when tested with the currently available assays.

## 5. The Influence of OBI on Development of HCC

It has been proposed that OBI is an important risk factor in the development of HCC [84] because it maintains the typical pro-oncogenic properties of an open HBV infection. Molecular and epidemiological studies in patients and in animal models have identified OBI as a risk factor for pre-neoplastic clonal expansion and development of HCC [85]. Although HBV is considered as a non-direct cytopathic virus, persistent and prolonged HBV infection in the liver leads to a slight but continual necro-inflammation mediated by the immune system that eventually contributes to the progression of cirrhosis and subsequent HCC [86]. The persistence of the virus in the liver may result in a slight but continuous necro-inflammation and could contribute over time to the progression of chronic damage to liver that causes cirrhosis [40].

The HBV genome has been detected in the tumor tissue of HBsAg negative patients with HCC, with a prevalence ranging from 30%–80% [87]. Moreover, a strong association has been reported between the presence of OBI in chronic hepatitis patients infected with HCV and the development of HCC when compared to patients infected with only HCV [88]. A recent study in Japan of a cohort of patients with cirrhosis non-related to HBV or HCV confirmed HBV DNA in the serum of patients with OBI as a predictor of a high rate of hepatocellular carcinogenesis. Eighty-two patients with cirrhosis who showed negative HBsAg and HCV levels were observed for an average of 5.8 years. The carcinogenesis rate was 27% in HBV-DNA positive patients and 11.8% in DNA negative patients at the end of the fifth year, and 100% and 17% in the 10th year, respectively [86]. Another study in Japan indicated OBI as a risk factor for development of HCC in non-cirrhotic patients after eradication of HCV by interferon treatment [89]. It is known that OBI decreases the response to interferon therapy when employed in patients with chronic hepatitis C and accelerates the progression of cirrhosis and HCC [90]. However, the presence of HCV in co-infection with HBV is not strictly necessary to favor the development of HCC. Despite all of these studies, it remains unclear whether the small amounts of HBV DNA found in people with OBI maintain their oncogenic potential in the absence of HCV [46].

In a study conducted in China, no HBsAg was detected in 70% of patients with HCC in the absence of chronic HCV infection [39]. In patients with cryptogenic HCC, the 73% (24 of 33 people) were found with OBI in at least two of four regions of the HBV genome, indicating that OBI is a probable risk factor in the development of HCC [91]. This study is of particular relevance because it used both tumoral and non-tumoral liver tissue for detecting OBI. Testing for OBI by only quantifying HBV DNA in the serum of these patients means that most individuals may be misdiagnosed as HBV-negative. Furthermore, it is well established that alcohol consumption causes cirrhosis and HCC; this study found that over 50% of patients with alcohol-related HCC were found to have OBI. This strengthens the possibility that patients with alcoholism may have an increased risk of synergistically developing HCC when they also present OBI.

Mutations in the HBV genome have also been associated with the development of HCC. In a study conducted in Taiwan, genetic variants were found in the Pre-S2 (M1I and Q2K) domains and in the enhancer II (G1721A) in the viral genome of people with HCC who carry OBI, compared to people with HCC but without HBV infection. This mutation pattern was proposed as a viral marker for HCC in people with OBI, which may aid in the identification of HBsAg negative cases with chronic progressive hepatitis and a high risk of developing HCC [92]. In a study conducted in six countries with different levels of endemicity for HBV, it has been suggested that HBV genotype C could play an important role in hepatocarcinogenesis in different geographic regions, and that genomic detection could be an essential factor in the clarification of unknown etiologic agents associated with HCC [93].

While there has been recent research related to this topic, controversy remains over the role of OBI in the development and progression of chronic liver disease and hepatic oncogenesis. It is therefore vital to use highly sensitive, specific and accurate diagnostic methods for OBI in order to conduct studies in different types of populations worldwide. Only then will it be possible to avoid unintentional bias in the analysis and finally establish whether OBI is a risk factor in the neoplastic transformation of the liver.

## 6. Appropriate Diagnosis of OBI

For the accurate diagnosis of OBI, it is necessary to use appropriate techniques to demonstrate the presence of HBV DNA in the host. Many people have been erroneously diagnosed with diseases or non-diagnosed when a more sensitive assay for the detection of HBV might have revealed another condition. The most sensitive current NAT tests detect OBI at higher rates than those used in previous generations. In many countries, NAT assays are routinely used in the management of HBV infections, especially to guide monitoring of the response to an antiviral therapy in chronically infected patients [94]. The gold standard test for OBI diagnosis is the HBV DNA in fresh liver tissue samples collected under conditions suitable for gene amplification procedures [24,70]. In fact, the time between the collection of biopsy and tissue freezing is a critical factor in the preservation and detection of specific viral nucleic acids, and should be less than three minutes [95]. The NAT assays must be conducted under conditions appropriate for the isolation of the viral genome. A feature of OBI is the presence of very low levels of the viral genome. The OBI detection range of these NAT assays is an average of 32–62 copies/mL or 5.10 IU/mL. However, most commercially available laboratory tests (Cobas Taqman 48 HBV, Cobas Amplicor HBV Monitor, NGI HBV SuperQuant, *etc.*) can only detect levels greater than 10^3^ copies/mL [46], implying that most cases of OBI are not diagnosed. The preferred lower limit of detection (LLOD) for HBV DNA, standardized by the World Health Organization (WHO), is ≤5 IU/mL or ~30 copies/mL [96]. It is therefore essential to adopt the most efficient procedures for the extraction of viral genetic material in order to ensure assay quality. There is a false OBI that is characterized by serum levels of HBV DNA comparable to those detected in an open HBV infection; however, DNA levels below 200 IU/mL are observed in true OBI [24]. Furthermore, the inclusion of appropriate controls for specificity and sensitivity is mandatory in each run of these tests while subsequent analysis of the sequence of the amplicons is recommended as a reinforcement measure. All assays directed toward the diagnosis of OBI should use primers containing at least three widely conserved regions of the HBV genome, such as the S, X and core genes [97]. Notwithstanding these international quality standards for the detection of the HBV genome, quantification by different assays still implies a wide and random variability. The main discrepancies in the results of OBI analysis are: (A) Different sample sizes in the studies; (B) The use of different laboratory equipment and measurement kits of different sensitivity and specificity; (C) Different racial, ethnic and genetic features that can affect humoral immune response to HBV, leading to differences in the production of anti-HBV antibodies [98].

## 7. Epidemiological Implications for Underdiagnosis of OBI in a Latin-American Region

A total of 20% of OBI cases are negative for all serological markers of HBV infection except DNA. This raises a very important scenario of underdiagnosis of OBI, especially in Latin American countries such as Mexico, where testing for hepatitis B is neither active nor systematic.

Mexico has been considered as a region of low endemicity for viral hepatitis B [99]. However, during the last 15 years there have been few studies that account for the prevalence of the HBsAg antigen and anti-HBc antibodies in the country. In this regard, there have been two national studies [100,101] with important methodological differences that restrict the direct comparison of their results [99]. The first investigates the seroprevalence of anti-HBc in some countries of Latin America. Its results place Mexico (with a seroprevalence of 1.4%) above Chile (0.6%), but below Argentina (2.1%), Venezuela (3.2%), Brazil (7.9%) and Dominican Republic (21.4%). The second study (Encuesta Nacional de Salud, ENSANUT 2000) reported a seroprevalence of HBsAg and anti-HBc of 0.21% and 3.3%, respectively [101]. These results allow us to estimate that in Mexico (over more than a decade), 1.7 million people contracted viral hepatitis B and at that there were at least 107,000 chronic carriers of the disease [101]. In the state of Veracruz, Mexico, which has one of the highest mortality rates of liver cancer in the country (2.56/100,000), no data have been published regarding the prevalence of hepatitis B. A study in apparently healthy donors found a seroprevalence of HBsAg of 0.057%, a value that is lower than that recorded nationally [102].

The prevalence reported above seems to be insignificant when compared with other regions of the continent or the world. However, there are reasons to believe that the true presence of HBV may be higher than the reported value [103]. These authors argue that there are a myriad of factors that contribute to hepatitis B presenting a much higher frequency than that reported. Aspects such as the presence of indigenous groups in Mexico, certain deficiencies in surveillance systems, vaccination programs focused primarily on hepatitis B in children that, for the moment, leave the adult population susceptible (until the vaccinated children can reach adulthood), and the use of serological tests of limited sensitivity and specificity [103].

Renal replacement therapy, particularly by hemodialysis, has been associated with a wide variation of occult hepatitis B infection [26,27,28,29,30,31,32]. For example, a very high prevalence has been reported in a hospital university in Spain (58%) [26], yet there is a complete absence of this condition in Italy [31] and Turkey [32]. As mentioned above, this may be due to multiple factors such as the sensitivity and specificity of the tests used to detect viral DNA, the inclusion criteria for study subjects, the particular medical care unit involved and the degree of endemicity of hepatitis B in the region of origin.

In Mexico, there are no official records of the number of patients nationwide who receive some form of dialysis treatment, and the prevalence of occult hepatitis B in these patients is therefore unknown. However, estimates suggest that such treatment is received by 510 people per million in Mexico, of which, 121 replacement therapy in renal function correspond to people on hemodialysis [104]. According to the results of the Population and Housing census 2010, which showed that more than 112 million people live in Mexico (precisely 112,336,538), it can be estimated that more than 13,000 renal failure cases exist that are currently undergoing hemodialysis treatment. If we consider the highest prevalence reported in Spain (58%) [26] as a worst-case scenario, in Mexico there may currently be more than 7000 cases of occult hepatitis B in renal failure cases only.

Another group at high risk for occult hepatitis B are those infected with HIV [33,34,35,36,37]. In Mexico, data from the ENSANUT 2000 show that the seroprevalence of HIV-I antibodies in people with age greater than or equal to 20 years is 0.25% [105]. Considering the fact that, according to the latest Census of Population, 67,397,224 people in Mexico are 20 years old or more, this would translate to 280,841 HIV-infected individuals in who OBI could be relatively frequent [106] by reactivation of latent anti-HBc due to their impaired immunological system [107]. The worldwide prevalence of OBI reported in this risk group ranges from 0–89.5%. This condition is of such importance that, in countries such as Cuba, modification of the search protocols for viral hepatitis B in HIV patients has been suggested [34]. In Mexico, there are no published studies in people with HIV, although such studies would be highly recommended in this group. With the above overview of the high-risk groups for OBI, the question arises of how the degree of endemicity of hepatitis B is correlated in different geographical regions with the prevalence of OBI and what is the nature of the mechanisms behind this. This could best be answered by the design and implementation of effective surveillance systems that identify hepatitis B in all its clinical variants.

As previously stated, OBI is usually not detected by the standard tests used in most developing countries. Mexico is included in this category and has no data on the prevalence of OBI in patients diagnosed with HCC that could help to clarify the cryptogenic condition. However, several studies in indigenous populations (Nahuas and Huicholes) [108] and in blood donors [109,110] have initiated research into OBI in Mexico.

There is a pressing need for the effective implementation of appropriate strategies and methodologies to detect HBV DNA, such as a worldwide screening test with greater emphasis on highly endemic regions and vulnerable groups. In Mexico, data on the prevalence of OBI are scarce due to the methodological techniques that are used for diagnosis. Current Mexican rules governing the use of human blood for transfusion requires the detection of HBV in each sample donor (NOM-253-SSA1-2012); however, DNA amplification tests are not used to detect HBV. Instead, immunosorbent assays that do not even consider HBcAg as a marker of infection are used as screening tests. A new immunoassay has already been developed that simultaneously detects PreS1 and core-related antigens, and even detects variants of HBsAg [111]. This test would be very useful for countries that do not implement NAT assays as screening tests. In order to standardize assays based on HBV DNA amplification, the WHO designed a standard code (code 97/750) with an output of 10^6^ IU (500,000 IU/vial) [96]. This highlights and emphasizes the appropriate implementation of molecular assays for the detection of HBV, taking into account the fact the low viral load in blood plasma (<200 IU/mL) [70] that is typical of OBI. The impact of the implementation of inadequate techniques for HBV diagnosis is the erroneous estimation of the number of carriers of the virus that could be far below the true figure and could thus seriously affect epidemiological surveillance warning systems. From a clinical point of view, this condition is extremely relevant because it may be the cause of liver disease under different scenarios not currently understood [25]. True improvement in conditions of health related to such infectious diseases will only be realized when national health systems worldwide can implement updated frontline methodological techniques. In the shorter term, studies that investigate the prevalence of OBI in patients with HCC will foster direct public support and design appropriate policies for the strengthening of comprehensive health systems.
